# “When am I going to stop taking the drug?” Enablers, barriers and processes of disclosure of HIV status by caregivers to adolescents in a rural district in Zambia

**DOI:** 10.1186/s12889-015-2372-3

**Published:** 2015-10-07

**Authors:** Mable Mweemba, Maurice M. Musheke, Charles Michelo, Hikabasa Halwiindi, Oliver Mweemba, Joseph M. Zulu

**Affiliations:** Department of Public Health, School of Medicine, University of Zambia, Lusaka, P.O. Box 50110, Zambia; Ministry of Community Development, Mother and Child Health, Lusaka, Zambia; Population Council, Lusaka, Zambia

## Abstract

**Background:**

Disclosure of adolescents’ own HIV status by caregivers is not only challenging but low. The reasons for this remain unclear despite efforts to examine and seek to understand disclosure patterns or factors that may either facilitate or inhibit this disclosure. This study explored the enablers, barriers and processes of disclosure of HIV status to adolescents by their caregivers in Kafue district of Zambia.

**Methods:**

A case study method was used to understand factors that facilitate or inhibit caregiver’s ability to disclose the HIV status of adolescents aged 10–15 years. Data collected through in-depth interviews with 30 caregivers as well as 6 key informants were analysed using thematic analysis.

**Results:**

Overall, 17 out of 30 (56.7 %) caregivers had informed the adolescents about their HIV status. Reasons for disclosing of the HIV status included inquiries by adolescents as to why they were taking medication, threats by adolescents not to take HIV medication, desire to promote treatment self-efficacy amongst adolescents as well as facilitating adoption of safe sexual behaviour among adolescents. The disclosure processes were conducted either at the home or at the clinic. Enabling factors for HIV disclosure were adolescents’ knowledge of HIV and caregivers’ knowledge of and experience with HIV programs. Barriers to disclosure of HIV status included fear of psychological trauma for the adolescents, perceived inability of adolescents to keep their HIV status confidential which could attract HIV stigmatisation for the family, and caregivers’, fear of being blamed by the adolescents for the infection, limited disclosure skills by caregivers as well as negative attitude by some HIV counsellors.

**Conclusions:**

Despite challenges associated with disclosure of adolescents’ own HIV status by caregivers, environments that facilitate this process exist and can be strengthened. Promoting HIV disclosure requires in-depth and context-specific understanding of the factors that enable and undermine this process. Limitations in this understanding may have played critical roles in past strategic implementation of locally driven and relevant interventions to improve disclosure of HIV status by caregivers to adolescents in Zambia.

## Background

Sub-Saharan Africa (SSA) remains the epicenter of the HIV epidemic with an estimated 2.5 million adults and children becoming infected in 2011, translating into 71 % of new global infections in adults and children [1]. Young people aged 15–24 accounted for 42 % of the new HIV infections [2]. Despite improvement in HIV testing, care, management and treatment processes, most people living with HIV, including adolescents, continue to maintain secrecy surrounding their HIV status [3]. Failure by caregivers to disclose to adolescents the HIV status of the adolescents negatively affects access to HIV care and treatment by adolescents living with HIV [4]. In Ethiopia, for example, the disclosure rate of HIV status was under 20 % [4, 5]. In west Africa, the disclosure rate of HIV status by caregivers to adolescents was 28.8 % [6]. A study in Ghana reported a disclosure rate of 21 % [7].

In most cases, disclosure by caregivers or parents ranges from being complete, partial, or not done at all [8]. Complete parental disclosure refers to a situation in which both the parent or caregiver and adolescent concur that the primary caregiver has told the adolescent about his or her HIV status and drugs which prevent opportunistic infections and help prolong life. Partial disclosure refers to a situation in which the adolescent is not fully aware of his or her HIV infection but is suspicious, asks the caregiver questions about the disease and the drug, and, in many cases, assumes that the drug is a cure. Non-disclosure is where the adolescent is unaware of his or her HIV status- [8].

Several factors affect disclosure of HIV status by caregivers to adolescents. These factors include fear of social exclusion or stigmatisation [8]. The caregiver may not inform the adolescent about his or her HIV status for fear that the adolescent may reveal the status to other community members thereby potentially attracting social stigmatisation [3]. Furthermore, the process of disclosing HIV status to adolescents may be inherently stressful [3]. In addition, some caregivers may not disclose the HIV status because they may lack adequate knowledge about the benefits of disclosure which include providing adolescents with sufficient time to grieve, an opportunity to talk openly about the diagnosis and the possibility of seeking clarifications from the mothers on any conceptions that the adolescents may have regarding HIV [9]. Limited skills by some caregivers on how to conduct HIV disclosure may also affect disclosure processes [10]. As a result, several studies have suggested that caregivers need support or capacity building on HIV disclosure processes [4–7].

Zambia has a prevalence rate of 13.3 % in the adult population aged 15–49 years [11]. Adolescents, defined as young people between 10–19 years of age constitute about 27 % of the Zambian population [12]. This group is not spared by the HIV epidemic. Estimates indicate that 80,000 of adolescents in Zambia are living with HIV [13]. For instance the recent Zambia Demographic and Health Survey (ZDHS) report indicates that 4.8 % of female and 4.1 % of male adolescents aged 15–19 years were living with HIV [11]. A study in Zambia showed that adolescents living with HIV who had not had their HIV status disclosed to them were younger and less likely to be receiving Antiretroviral treatment [14].

While disclosure of HIV status to adolescents is critical to facilitate access to continuum of HIV care, there still exists limited knowledge on the factors that promote or hinder disclosure of HIV status by caregivers to adolescents living with HIV. Most of the studies conducted in Africa on HIV disclosure patterns have not focused on this age group [4–6]. This study therefore aimed to contribute towards reducing this knowledge gap by exploring factors that enable and undermine caregiver’s ability to inform adolescents of their HIV status in Kafue district, Zambia.

## Methods

### Study design

A qualitative case study methodology was used to explore issues that affect caregivers’ ability to disclose or inform the adolescents of their HIV status. The case study methodology is an empirical approach that investigates contemporary phenomena within a real-life context, where the boundaries between phenomena and context are not clearly evident and in which multiple sources of evidence are used [15]. The case study approach was considered appropriate for this study because the caregivers and adolescents live within a complex context, which involves social interactions. Using this approach enabled us to capture the social and cultural realities of the context which shape disclosure processes.

### Sample size and sampling procedure

The study was conducted in Kafue district, located about 45 kilometres south of Lusaka. The setting was purposively selected because it is one of the districts with the highest HIV infection rates in Zambia. This district is predominantly rural, although a smaller segment of the population is urban-based. The people in the district are involved in different kinds of livelihood activities mainly in the informal sector, such as small scale farming, selling farm products in markets, fishing activities and running of small retail shops. There are a small proportion of people in formal employment, mainly government workers.

Three facilities were purposefully selected for the study because they provide complete antiretroviral services in the district. These facilities are Nangongwe clinic, Estates clinic and Kafue district hospital. The study population comprised caregivers of adolescents who were in the HIV care program, regardless of whether they were on antiretroviral therapy on not. These were recruited from the three health facilities using clinical records. Lay HIV counsellors, peer educators or nurses directly involved in the provision of HIV care to adolescents in the selected paediatric clinics were also interviewed.

There were 60 adolescents in the HIV care program from the three selected heath facilities, and also 60 caregivers. All these 60 caregivers were eligible for the study. Thirty (30) caregivers (about 10 per health facility) were purposively recruited using a maximum-variation sampling criteria from the three selected health facilities. Maximum variation sampling involves selection of study participants to reflect the diverse characteristics of the study participants, in this case the age of the adolescents, gender, length of time on treatment (if on medication) and relationship between adolescent and the caregiver. Thus, young caregivers like brothers or sisters, old caregivers such as grandparents, uncles and aunties and actual parents helped generate in-depth unique insights and shared patterns of issues that shape HIV disclosure that cut across cases. Caregivers whose adolescents knew their HIV status before they started providing care to them were excluded from the study because they would not have had the experience of disclosing HIV status to the adolescents. The caregivers who were taking care of adolescents aged 10–15 years living with HIV and agreed to participate in the study by signing a written informed consent were enrolled for the study. All lay counsellors, peer educators and nurses in ART facilities that were directly involved in HIV service delivery to adolescents were enrolled in the study as key informants. A total of six (6) key informants were interviewed, 2 from each facility.

### Data collection techniques

#### In-depth interviews with caregivers

Thirty (30) in-depth interviews with care-givers were conducted by the first author, who has experience in qualitative research. These interviews were conducted both in English and the local language (Tonga) using an interview guide designed for this purpose. Data collection was done concurrently at the three selected health facilities because the clinics have different days in which they attend to adolescents living with HIV. Data collected included: socio-demographic characteristics of respondents, disclosure status of the adolescents, age at disclosure and how caregivers informed adolescents about their HIV status.

#### Key Informant Interviews with health facility staff

Six (6) key informant interviews were conducted with health care providers. The interviews were conducted in English as the respondents were conversant with the language. The interviews aimed at triangulating the issues raised by the care providers during in-depth interviews and understanding health providers’ experience with disclosure and suggestions for improving disclosure.

### Data analysis

All interviews were recorded digitally and later transcribed verbatim by the first author and reviewed by all authors. Interviews conducted in the local language were translated into English. The transcripts were stored on a password-protected computer with access only restricted to the authors. The interview transcripts were then entered into QSR NVivo version 10. Latent content analysis was used to analyze and interpret the data. This analysis processes first involved reading the data several times to create an understanding of the whole data set [16]. The interview transcripts were then coded, and the codes were compared for similarities and differences by conducting within-and across-case analysis [17]. Similar codes were then grouped to form categories and finally themes were developed by interpreting the categories for their underlying meaning. The codes, categories and themes were separately reviewed by the authors in order to enhance validity of the findings.

### Ethics

The study was approved by the ERES CONVERGE Independent Review Board, one of the local Research Ethics Committees in Zambia (Reference number is 2013-Aug-002). Written informed consent was obtained from all study participants. To avoid involuntary disclosure of HIV status to the adolescents, the interviews were conducted in the absence of the adolescents. The interviews were conducted in private spaces within the health facilities to ensure confidentiality. In order to ensure participants’ confidentiality, no names or personal identifiers were included in the transcripts. All the respondents were assigned numerical codes. There was no physical harm to the participants as the study did not involve administration of invasive medical instruments. However, the study could have posed minimal psychological harm as evidenced by the experiences where caregivers, especially biological parents ended up disclosing their own status in the process of the interview. Confidentiality was reassured and maintained by keeping the process as private as possible. Counselling services were offered in view of the above as need arose. The participants were not given any direct immediate benefits as they were being interviewed within the clinic environment and at the time that they brought the adolescents for medical attention.

## Results

### Characteristics of the caregivers and adolescents

Thirty (30) caregivers took part in the study. Half (15) of them were biological parents, two (2) guardians were foster parents and thirteen (13) were relatives to the adolescents. Twenty-three (23) of the caregivers were female (Table 1). In terms of how the caregivers came to know about the HIV status of the adolescents, fourteen (14) took the adolescents for HIV testing themselves. Fourteen (14) of the adolescents had been tested for HIV between 2 and 5 years ago; ten (10) had been tested for HIV less than 2 years ago.

**Table 1:** Characteristics of the caregivers

Type of caregiver	Gender of caregivers		Total
	Female	Male	
Biological parent	11	4	15
Foster parent	2	0	2
Relative	10	3	13
Total	23	7	30

The thirty adolescents whose guardians were interviewed were aged between 10 and 15 years, and the majority of the adolescents were aged 11 years old (Table 2). Male adolescents were twenty (20) while ten (10) were female. All the adolescents were on antiretroviral treatment (Table 2).

**Table 2:** Adolescent who knew their HIV status by Age and Sex

Age of Adolescents	Know HIV status		Do not know HIV status		Total
	Male	Female	Male	Female	
10 years	2	0	3	1	6
11 years	5	1	3	2	11
12 years	2	0	0	0	2
13 years	1	1	1	1	4
14 years	1	3	1	0	5
15 years	0	1	1	0	2
Total	11	6	9	4	30

### Enablers of HIV disclosure to adolescents

Slightly over half of the caregivers had disclosed the HIV status to the adolescents (17 out of 30). The male adolescents that did not know their HIV status were about twice the number of female adolescents that did not know their HIV status (9 against 4). Equally, the number of males that knew their HIV-positive status was almost twice that of females (see Table 2). The reasons for disclosure were varied and these are described below.

#### Inquiries by adolescents

One of the recurring reasons why caregivers informed adolescents about their HIV status was due to persistent inquiries by the adolescents as to why they were on medication despite not being ‘sick’. The adolescents would ask how long they had to continue taking the drugs. On this basis, caregivers used this as a window of opportunity to inform adolescents about their HIV status:

‘When am I going to stop taking the drug’? The child asked me that is when I explained to him that this medicine you will drink forever. The medicine to cure this disease has not yet been found and if you stop, you will be sick. (45-year old male caregiver)

#### Adherence to treatment

Another reason for informing the adolescents about their HIV status was poor adherence to HIV treatment by some adolescents. Interviews with caregivers showed that some adolescents did not regularly take their medication and at times threatened to stop taking medication. Therefore, in an attempt to promote adherence to medication, caregivers informed adolescents of their HIV status. One 50-year old male caregiver explained this view in the following way:

I told him when he developed a habit of refusing to take his medication; so when I came here they (clinic staff) told me that I have to disclose to the adolescent exactly why he is taking the drugs; he has to know the reason why.

#### Independent access to HIV care

Some caregivers also reported informing adolescents about their HIV status on the advice of health care providers in order to promote self-efficacy among adolescents. This was aimed at facilitating the ability of adolescents to go to the health facility unaccompanied instead of relying on caregivers who may not always be available. A 50-year old female caregiver explained:

I disclosed because we were being advised to do so at the hospital. We were told to start disclosing because these adolescents are growing up and soon they will become independent and will be going to the hospital alone and they will not be able to answer questions on their own.

#### Trust between caregiver and adolescent

Maintaining future cordial relationships between the caregivers and adolescents was another reason for disclosing HIV status. Some respondents narrated that they had been advised by health care providers that it was important for them as caregivers to inform adolescents of their status as opposed to them knowing of their HIV status from the clinic to promote trust.

#### Adoption of safe sexual behaviour

Facilitating adoption of safe sexual behaviour was another reason for disclosing HIV status. Caregivers were aware that as adolescents grew into adulthood, they became sexually active. Thus informing them of their HIV status was regarded as important to enable them adopt appropriate sexual behaviour practices to avoid re-infection or infecting their sexual partners.

### HIV disclosure locations and processes

Disclosure of the HIV status by caregivers to adolescents was done either at home or at the health facility. It was reported that this disclosure was an on-going process. The adolescents needed continued learning about what it meant to live with HIV till they had full understanding of their HIV condition. Health care providers explained that the disclosure process with some adolescents took a long time, and that there was a need to plan for this long and gradual disclosure process.

#### Direct conversation between caregiver and adolescent at home

Disclosure through direct conversation between the caregiver and the adolescent was often triggered by questions being asked by the latter about their sickness or medications they were taking every day even when they did not feel sick. Inquisitiveness about their HIV status was also facilitated by the knowledge which they acquire through health education at the health facility. In most cases, the caregiver would disclose because they would want the adolescent to get the information about their HIV-positive status from them and not from anyone else or the clinic. Sometimes disclosure happened when the caregiver was alone with the adolescent or in the presence of some important family members:

I told him about his HIV status when we were together at home because he was asking that “when am I going to stop taking the drugs”? (45-year old female caregiver)

Interviews with health care providers, however, showed that direct conversations between the caregivers and the adolescents about their HIV status also involved caregivers disclosing their own HIV-positive status too. Thus, the fear of disclosing their own HIV status also turned out to be a barrier to caregivers’ ability to inform adolescents their HIV status. They also noted that the disclosure process required thinking about ways of mitigating possible negative reactions from the adolescents involved by assuring them about a positive future, including the possibility of an HIV cure.

#### Assisted disclosure at the health facility

While some caregivers were able to directly disclose to the adolescents, other caregivers would fail to do so. As a result, they reported asking for help from health facility staff to assist with the disclosure process. At the health facility, disclosure followed a systematic process. In most cases, this process often started with counselling for the caregiver. Caregivers were counselled in order to build their capacity so that they could effectively handle the disclosure process. Then, the health facility counsellor would counsel the adolescent separately and later on together with the caregiver before disclosing HIV status to the adolescent.

Disclosure processes were largely dictated by experiential or information-based constructed knowledge levels. First, adolescent knowledge of HIV facilitated the disclosure process. Caregivers reported that disclosure process was easier when adolescents had adequate knowledge on the relevance of HIV testing, taking and adhering to antiretroviral treatment. Second, questions by adolescents regarding uptake of medication every day was also used by caregivers as a platform to inform adolescents of their HIV status. Third, caregivers’ knowledge of and experience with HIV also positively shaped HIV disclosure. For some caregivers, their knowledge on HIV and work experience in HIV programs, either as formal or informal health care workers, was useful in ensuring that they had the requisite skills and were comfortable to inform adolescents their HIV status. One caregiver who also worked as an HIV counsellor explained:

Because of my involvement in HIV programmes, I learnt about importance of making adolescents aware of their HIV status. I leant that HIV status awareness helps in promoting good adherence to treatment and planning for the future. It also helps preventing spreading of the infection or being infected. This knowledge helped me to effectively disclose HIV status to my adolescent.

### Barriers to disclosure of HIV status

#### Age of adolescent

Young age of the adolescents was cited as one of the barriers to HIV status disclosure process. Most of the caregivers that had not yet disclosed and even those that did partially disclosed described the young age of the adolescents as the underlying reasons for not informing them of their HIV status. Caregivers feared that some of the adolescents were not mature enough to be able to adequately grasp issues.

#### Adolescent exposure to psychological trauma

Another barrier to HIV disclosure was fear of psychological trauma: It was feared that informing adolescents about their HIV status could make some of the adolescents psychologically traumatised. It was further feared that such trauma could result in depression or into behaviours that may be harmful to adolescents or to other people:

We fear to tell them about their HIV status. It is because some adolescents would want to commit suicide due to unstable emotions or short tempers; because they think they are already dead…some adolescents even become furious and violent. (50-year old male caregiver)

#### HIV stigma

In addition, this study also found evidence showing that HIV stigmatisation affected HIV disclosure process. Some caregivers could not tell adolescents about their HIV-positive status for fear of exposing them to stigma in the event that their peers came to know about their HIV status.

#### Protection of caregiver reputation

Other caregivers reported being reluctant to inform adolescents of their HIV status in order to protect their own reputation in instances where the caregiver was also HIV positive. This was particularly the case in instances where the adolescent had acquired HIV through mother-to-child transmission. One health care provider put it this way:

May be they are scared of being embarrassed. In my own thinking maybe it is because they fear to be embarrassed, they fear that the child will say it is them who caused the child to be infected.

#### Blame for HIV infection

The other factors that limited HIV disclosure included fear of being blamed for the HIV status. Fear of being blamed for causing HIV infection through mother-to-child transmission of HIV was one of the major reasons why some caregivers were reluctant to inform adolescents of their HIV status. This was expressed by both caregivers and health care providers. One health care provider explained: *“Hatred, fear, especially to parents, both parents who are alive, they fear. They fear to be blamed….”*

#### Disclosure skills

In some instances, caregivers also acknowledge their lack of disclosure skills as the reason for not informing adolescents of their HIV status. Health care providers confirmed that most caregivers lacked skills on how to go about the disclosure process. It was reported that most caregivers did not know what and how to tell the adolescents regarding their HIV status:

I don’t know…maybe it is not knowing or not getting proper guidance on how to go about it because imagine if you knew how to say it, it would even take 24 h from the time you were told, you can just do it there and then. But it becomes…eeh, how am I going to tell her? Where do I start from? (42-year old female caregiver)

#### Attitude of HIV counsellors

Negative attitude by some HIV counsellors was another barrier to HIV disclosure. Even though caregivers reported being assisted by care providers to disclose the HIV status to the adolescents, some caregivers described some counsellors as not helpful, thus undermining efforts to seek help from them. One 42-year old female caregiver narrated her experience with a counsellor as follows:

People are not the same here… there are some counsellors who are good… they would counsel you, they would counsel the adolescent, and even if it is not you…. But others are not good. It’s like…the way they talk to you or the adolescent…. It is not encouraging.

## Discussion

This study has demonstrated that there are factor that shape caregivers’ disclosure or non-disclosure of HIV-positive status to adolescents under their care. These factors included caregivers’ weighing of the benefits of disclosure against the social-psychological dangers, levels of knowledge by caregivers about disclosure strategies, age of adolescents, and assessment of social and psychological impact of awareness of the adolescent of the HIV status as well as support from the health care providers.

The findings also suggest that disclosure is not a straightforward, linear process; rather it is a complex, interlinked, non-linear and dynamic process driven by contexts and constructs at both individual and community level. Disclosure in most cases is not a one-off event, but a process which is influenced by the social context, the perceived appropriate age for disclosure of HIV status, the perceived maturity level of the adolescent to handle HIV information, and the role of health care providers in facilitating disclosure. It is also influenced by fears of backlash from the adolescents, the fear of exposing adolescents to psychological trauma, and the perceived level of stigma in the social context. On the disclosure process, our findings therefore seem to appear to contradict the four stages of disclosure put forward by Tasker [18]. These stages comprise, a) secrecy stage, where parents want to keep all knowledge about the illness from the adolescent; b) exploratory stage, where they will begin to give some explanations to their adolescent; c) readiness stage, when they give further information and prepare more fully; and lastly, the disclosure stage, when the adolescent is told the name of the virus [18]. Because disclosure is not a straightforward process, adequately facilitating the process demands that caregivers should have adequate skills to do so. The findings however suggest that caregivers encounter challenges in disclosing the HIV status to the adolescents, namely “the when”, “the how”, and “what to inform” adolescents about their status. This is further complicated by instances where caregivers are themselves HIV-positive and fear being blamed by the adolescents for infecting them with an incurable infection. While requiring statistical proof through other studies, these findings may suggest that caregivers who could have infected adolescents through mother-to-child transmission are unlikely to inform adolescents of their HIV-positive status. Our findings are similar to other studies who found that fear of being judged and blamed by the adolescent dissuaded caregivers from telling adolescents their HIV-positive status [8, 10, 19]. Furthermore, this may point to a need for closer rapport between service providers and the guardians to identify the best possible options and processes of disclosing HIV status to the adolescents [4].

This study also suggests that barriers to disclosure of HIV status to adolescents are not mutually exclusive. They are interrelated and some may intersect and coalesce to undermine disclosure. For instance, while perception of young age of the adolescent reduces motivation to inform the adolescent of his/her HIV status, fears about stigma, sometimes influenced by guardians attempt to preserve their own social image, may undermine disclosure of HIV status to adolescents. Similarly, although lack of disclosure skills may undermine disclosure of HIV status to adolescents, concerns about young age of adolescents also dissuade caregivers from telling adolescents their HIV-positive status. Therefore, the inter-sectionality of these findings make one point saliently clear: there is no single barrier to disclosure of HIV status to adolescents. These findings point to a need for a multi-pronged approach to addressing these barriers.

Further the findings of this study, like other studies, suggest that caregivers weigh the benefits of disclosure against the social-psychological dangers [4, 7, 9, 10, 20–22]. The findings of this study also suggest that caregivers’ decisions to disclose are influenced by their concerns around treatment adherence, the eminent onset of sexual activity of adolescents, and their desire to protect their adolescents, and to protect others from being infected. The findings are consistent with the findings of Vaz et al. [19] who reported the need to facilitate adherence to treatment and to live a healthy lifestyle as underlying reasons for disclosure of HIV status. However, disclosure of HIV status is undermined by fear that knowledge of HIV status would traumatize the adolescent. Evidence elsewhere, however, shows that these fears are unwarranted. The study conducted in the United States of America by Santamaria et al. [21] found that disclosure and timing of disclosure were not significantly associated with negative psychological functioning. Compared with adolescents who had not been told their HIV status, adolescents who knew their HIV status reported significantly less anxiety. These findings appear to contradict those of Vaz et al. [19]. In their study conducted in the Democratic Republic of Congo, they found that whilst the adolescent did not feel angry or anxious upon learning their HIV status, they, however, reported feeling either sad or heartbroken, worried, afraid and frightened [19].

The findings of the study also indicate that the fear of exposing adolescents to stigma and discrimination undermine efforts to tell them of their HIV-positive status. These findings are consistent with the studies which reported fear of discrimination, social rejection and isolation as barriers to adolescent knowledge of their HIV status [19, 23–27].

In general, these findings suggest that use of blanket approaches in implementing strategies for enabling disclosure of HIV status by caregivers to adolescents may not be effective. Instead, disclosure should be implemented taking into account the unique characteristics of the adolescent, including the availability of social support and perceived level of stigma in the community. This is because encouraging disclosure in the quest to facilitate adherence to treatment may be achieved at the expense of adolescent-peer relationships, especially in an unsupportive and highly stigmatizing social environment. Further, it is also important to design strategies for improving disclosure skills of caregivers, promoting involvement of health providers in disclosure processes, establishing adolescent-specific clinic days which could allow adolescents to mingle with their friends and learn from them, as well as increasing efforts for reducing stigma within the community.

### Limitations and strengths

A more general limitation of this study concerns the generalisability of the findings. This study was conducted in one setting with a small sample of respondents drawn from three (3) health facilities of Kafue district. The findings may therefore not be representative of other settings given the design chosen. Similar studies are therefore warranted in other settings for comparability of findings. Another limitation relates to the age category of adolescents whose caregivers were interviewed. The majority of them were around 11 years of age. It is possible that the perceived young age of the adolescents could have led to caregiver’s reluctance to inform them of their HIV status. Studies targeting older adolescents (15–19 years old) are warranted for comparability of findings. Further, the study did not explore the adolescents’ level of cognitive development which is important because available evidence suggest that neurological and cognitive function of perinatally-infected children is delayed and impaired as a result of HIV infection [28, 29]. Our study suggests that caregivers used their own lay assessment of the maturity of the adolescents as a basis of disclosing HIV status to them, a factor that could be plagued with inherent intra-observer biases. In addition, while the findings of this study suggest that the quest to ensure adherence was one of the reasons for disclosure of HIV status, it was beyond the scope of this study to establish the relationship between disclosure of HIV status and adolescent adherence to treatment. Future studies should therefore explore this relationship. Notwithstanding the limitations, through maximum variation sampling of study participants, the strength of this study was the diversity of the guardians of the adolescents –biological parents, foster parents and other caregivers, as well as representation of the age categories of the adolescents. Therefore, the findings probably provide analytical generalizations that can apply to other similar settings.

## Conclusions

We conclude that the observed disclosure determinants of HIV status from caregivers to adolescents in these informants suggest that differential disclosure patterns are multi-factorial in nature. This is unlikely to be due to either observational bias driven by an occurrence at a point in time, rather it might be suggestive of omnipresent social status and not peculiar to the community the informants were derived from. This may also define disclosure patterns for other similar disease domains. The findings suggest that disclosure rate is low, and it is mostly influenced by desire to enhance adherence to treatment and ensure adoption of safe sexual behaviour. Improved level of disclosure is hampered by the perceived young age of the adolescents; fear that knowledge of HIV status would traumatize the adolescent, fear of being blamed for HIV infection, and lack of disclosure skills. These findings underscore the need for in-depth, context-specific understanding of the hurdles to disclosure and the need to implement locally relevant, needs-based and beneficiary responsive intervention programmes to promote disclosure. This is because, as pediatric HIV scale-up continues, the low rates of disclosure may undermine the gains being scored in scaling-up access in resource-limited settings such as Zambia.
